# Prevalence of Thyroid Dysfunction among Patients with Type II diabetes Mellitus in Tertiary Care Center: A Cross-sectional Descriptive Study

**DOI:** 10.31729/jnma.8787

**Published:** 2024-10-31

**Authors:** Lokraj Kandel, Yagya Laxmi Shakya, Manish Yadav, Newton Ashish Shah, Sanjay Gupta

**Affiliations:** 1 Department of General Practice, Maharajgunj Medical Campus, Institute of Medicine, Nepal; 2 Tribhuvan University Teaching Hospital, Maharajgunj, Kathmandu, Nepal

**Keywords:** *diabetes mellitus*, *glycemic control*, *type 2*, *thyroid diseases*

## Abstract

**Introduction:**

Diabetes mellitus (DM) and thyroid dysfunction (TD) are two of the most prevalent endocrine disorders globally. Both conditions have significant effects on metabolism and are known to influence each other. Thyroid hormones play a crucial role in glucose homeostasis by affecting insulin secretion, sensitivity, and glucose metabolism. Conversely, altered glucose metabolism in diabetes can impact thyroid function, leading to a higher prevalence of thyroid disorders in diabetic patients. Understanding this relationship is important for improving the clinical management of patients with Type II DM. However, there is limited data on the prevalence of thyroid dysfunction in diabetic populations within specific regions.

**Methods:**

A cross-sectional descriptive study was conducted from February to September 2023 among 209 patients diagnosed with Type II DM at a tertiary care center after obtaining ethical approval from Institutional Review Committee(Reference No: 367 (6-11) E2). Thyroid function was assessed using thyroid-stimulating hormone (TSH), free T3 (FT3), and free T4 (FT4) levels. Thyroid dysfunction was classified into hypothyroidism and hyperthyroidism based on standard clinical and laboratory criteria. Data management involved entry into Microsoft Excel, verification for accuracy, and subsequent analysis using SPSS version 29.

**Results:**

The study included 209 patients with 128 (61.24%) females and a mean age of 65.87±13.7 years. The prevalence of thyroid disorders was 77 (36.84%) 53(25.36%) hypothyroidism,24 (11.48%) hyperthyroidism among patient with type II diabetes mellitus.

**Conclusions:**

Our study shows a high prevalence of thyroid disorders, especially subclinical hypothyroidism, in individuals with type 2 diabetes. Poorly controlled blood glucose (HbAlc > 7.5) significantly increases the risk, underscoring the need for routine thyroid screening in T2DM management

## INTRODUCTION

Type 2 diabetes mellitus (T2DM) and thyroid disorders (TD) represent significant challenges in contemporary healthcare, affecting millions worldwide and posing complex interactions that warrant deeper investigation.^1^ T2DM affects approximately 415 million adults globally and is a critical public health concern due to its associated complications and economic burden.^2^ In parallel, thyroid disorders encompass a spectrum of conditions, including hypothyroidism and hyperthyroidism, impacting thyroid hormone production and metabolism regulation, crucial for overall health.^3^

The interplay between T2DM and thyroid dysfunction has been extensively studied since its initial documentation in 1979, revealing intriguing connections and mutual influences.^4,5^ Shared risk factors such as obesity underscore the complex relationship between these disorders, influencing disease onset and progression.^6^ Notably, thyroid hormones play pivotal roles in regulating glucose metabolism, suggesting that even subtle variations within normal thyroid hormone levels could predispose individuals to insulin resistance and T2DM, particularly in susceptible populations.^7,8^

Recent research has highlighted a bidirectional relationship between T2DM and thyroid disorders, emphasizing their intertwined pathophysiology and clinical implications. Studies indicate that T2DM may influence thyroid function through mechanisms such as altered thyroid hormone synthesis and secretion.^9,10^ Conversely, thyroid disorders, especially hypothyroidism, can exacerbate insulin resistance and glucose intolerance, complicating diabetes management^10^ These findings underscore the need for a comprehensive understanding of how these conditions interact to optimize patient care strategies. This study aims to assess the prevalence of thyroid dysf unction,These insights can improve clinical practices and outcomes for T2DM patients with thyroid dysfunction.

## METHODS

This prospective cross-sectional observational study was conducted at the General Practice Outpatient Department (GP OPD) and General Health Checkup (GHC) clinic of Tribhuwan University Teaching Hospital from February 1, 2023 to September 1, 2023. It utilized quantitative research methods and employed a nonprobability sampling technique. Ethical approval was obtained from the Institutional Review Board (IRB) with a reference no. 367 (6-11) E2 of the Institute of Medicine (IOM), Maharajgunj, Kathmandu prior to study commencement. A total of 209 patients were included based on calculated sample size requirements, considering an expected proportion of thyroid disorder prevalence in T2DM patients and a precision level for the estimate. Criteria were established for participant selection, including inclusion of diagnosed T2DM cases aged 40 years or older without history of thyroid surgery, trauma to the neck, or exposure to neck radiation. Exclusion criteria encompassed patients under 40 years old at T2DM diagnosis, those with relevant medical histories, pregnant women, and users of specific medications affecting thyroid function. Data collection utilized a structured case proforma capturing socio-demographic parameters, physical examinations, and clinical parameters, with thyroid function tests conducted at the hospital's Biochemistry Lab. Data management involved entry into Microsoft Excel, verification for accuracy, and subsequent analysis using SPSS version 29. This approach aimed to provide comprehensive insights into the correlation between T2DM and thyroid disorders, contributing to enhanced clinical practices and healthcare strategies.

The sample size was calculated using the formula as follows:


$$\begin{array}{ll} \text{n} & ={\text{Z}}^{2}\times \frac{\text{p}\times \text{q}}{{\text{e}}^{\text{2}}}\\ & =1.96^{2}\times \frac{\text{0.162}(\text{1}-\text{0.162})}{0.05^{2}}\\ & =209 \end{array}$$

Where:

n is the minimum required sample size for an infinite population.

z represents the z-value (1.96) at a 95% Confidence Interval (CI).

p is the expected proportion of thyroid disorders in Type II diabetes mellitus based on previous studies (0.162)^11^

q = 1-p

e is the margin of error taken as 5% of estimated

Applying these values, the calculated sample size was approximately 209. A non-probability convenience sampling method was used for this study.

## RESULTS

This study was conducted at the General Outpatient Department (GPOPD) and General Health Care (GHC) clinic of a tertiary care health center from February 1, 2023, to September 1, 2023, involving patients diagnosed with type 2 diabetes mellitus (T2DM). A total of 219 cases were screened for eligibility over the 7-month study period. Of these, 7 cases were excluded, and 3 patients refused to provide consent. Among the excluded cases, 5 had known thyroid disorders, and 2 had a history of thyroid surgery. Consequently, 209 cases met the inclusion criteria and were included in the study. The mean age of the patients was 65.87±13.7 years with 128 (61.24%) females. Patients were mostly from Bagmati Province 85 (40.67%), followed by Madesh Province 32 (15.31%), with the fewest patients from Karnali Province 8 (3.83%). According to the Body Mass Index (BMI) classification for Asians (NICE guidelines, 2013), most patients were overweight 111 (53.11%), followed by obese 74 (35.41%), with the least being underweight 5 (2.39%) (Table 1). Based on HbA1c levels, patients were divided into two groups: A) Group 1: Controlled blood glucose levels (HbA1c < 7.5). B) Group 2: Uncontrolled blood glucose levels (HbA1c ≥ 7.5).

Among the 209 T2DM cases, 77 (36.84%) had thyroid disorders, with 53 (25.36%) cases of hypothyroidism and 24 (11.48%) of hyperthyroidism. The prevalence of thyroid disorders in T2DM patients was 77 (36.84%), with subclinical hypothyroidism being the most common. Total hypothyroid cases were 53 (25.36%) of which 19 (35.85%) cases were in the controlled diabetes group (HbA1c < 7.5) and 34 (64.15%) cases were in the uncontrolled diabetes group (HbA1c ≥ 7.5). Similarly, 24 (11.48%) patients were hyperthyroid out of which 8 (33.34%) cases were in the controlled diabetes group (HbA1c < 7.5). And rest 16 (66.67%) cases were in the uncontrolled diabetes group (HbA1c ≥ 7.5) (Table 2).

**Table 1:** Demographic characteristics of the study population. (n=209)

Variables	n (%)
**Sex**	
Female	128 (61.24)
Male	81 (38.76)
**Age**	
40-49	31 (14.83)
50-59	39 (18.66)
60-69	56 (26.79)
70-79	43 (20.57)
80-89	32 (15.31)
90-94	8 (3.83)
Age mean (SD)	65.87±13.7
**Address**	
Bagmati	85 (40.67)
Far western	15 (7.18)
Karnali	8 (3.83)
Gandaki	25 (11.96)
Koshi	23 (11)
Lumbini	21 (10.05)
Madhesh	32 (15.31)
**BMI**	
Underweight <18.5	5 (2.39)
Normal 18.5 - 22.9	28 (13.4)
Over Weight 23-27.4	111 (53.11)
Obese >= 27.5	74 (35.41)

**Table 2:** Distribution of thyroid dysfunction by HbA1c levels in Type II diabetes patients.

Thyroid Disorder	HbA1c < 7.5 (Clinical)	HbA1c < 7.5 (Sub clinical)	HbA1c ≥ 7.5 (Clinical)	HbA1c ≥ 7.5 (Sub clinical)
Hypo thyroidism (n=53)	6 (11.32)	13 (24.53)	12 (22.64)	22 (41.51)
Hyper thyroidism (n=24)	1 (4.17)	7 (29.17)	6 (25)	10 (41.67)

## DISCUSSION

Our findings on the prevalence of thyroid disorders among patients with type 2 diabetes mellitus (T2DM) align with and expand upon existing literature. Specifically, our study observed a prevalence rate of 36.84% for thyroid disorders within the T2DM cohort studied at Tribhuwan University Teaching Hospital, which is higher than rates reported in some previous studies. For instance, Feely et al. reported a prevalence of 18.5%,^12^ Pasupathi et al. found 16.4%,^13^and Pimenta et al. reported 26.7%.^14^ Conversely, our prevalence was lower compared to Udiong et al., who reported a prevalence of 46% in a similar demographic.^15^ Notably, our study also corroborates findings by Hollowell et al., who reported a prevalence of 35%, indicating consistency across diverse populations.^16^

Demographically, our study population predominantly consisted of females 61.24% with an age range from 40 to 94 years, similar to findings by the Diabcare Nigeria study group, which reported a 60.5% female preponderance.^17^ Geographically, most of our patients were from the Bagmati province 40.67%, followed by the Madesh province 15.31%, and the least from Karnali province 3.83%. These demographic pattern highlights the regional variations that may influence the prevalence and clinical management of thyroid disorders in T2DM patients.

The high prevalence of thyroid disorders among patients with type 2 diabetes mellitus (T2DM), as observed in our study 36.84%, highlights critical implications for clinical practice. Integrating routine thyroid screening into T2DM management protocols is crucial to early detection and tailored treatment strategies.^18^ This approach not only enhances patient care by addressing potential complications early but also underscores the interplay between thyroid function and glycemic control. Our findings favor comprehensive care models that consider both endocrine conditions concurrently, aiming to optimize therapeutic outcomes and mitigate associated risks, such as cardiovascular complications. Moreover, assessing whether these prevalence rates align with global trends or are influenced by regional factors allows for more targeted interventions that meet specific patient needs, thereby improving overall health outcomes in T2DM populations.

Existing literature suggests that insulin resistance in type 2 diabetes mellitus (T2DM) may disrupt thyroid hormone metabolism, leading to alterations in TSH secretion and subsequent thyroid hormone levels^19^. In our study we found that 25.36% of diabetic patients were suffering from hypothyroidism. It is known that insulin an anabolic hormone which inhibits the hepatic conversion of T4-T3, enhances the levels of FT4 while it suppresses the levels of T3,^15,21^ that lead to increase level if TSH. Hypothyroidism falsely raises HbA1c due to decreased erythropoiesis.^21^ Poor glycemic control (increased HbA1c) can be directly linked to the development of thyroid dysfunction in T2DM. This finding is similar to previous studies done by SU Ogbonna et al.^23^ Papazafiropoulou A et al.,^21^ Sathish R et al.^24^ Elevated HbA1c levels could impact thyroid hormone synthesis and secretion pathways, influencing TSH production and thyroid hormone bioavailability. With the potential to inform novel therapeutic strategies targeting both diabetes and thyroid disorders simultaneously, these findings highlight the need for additional research to clarify the precise pathways involved.

Our study design possesses several strengths that enhance the robustness of our findings. The utilization of a cross-sectional observational approach allowed for the comprehensive assessment of thyroid disorders in a sizable cohort of type 2 diabetes mellitus (T2DM) patients within a defined timeframe. The study was conducted at a tertiary care center known for its diverse patient population, ensuring a broad representation of demographic and clinical characteristics. The adoption of rigorous inclusion and exclusion criteria minimized confounding variables, thereby enhancing the internal validity of our results. Additionally, the use of standardized data collection techniques and validated measurement tools, such as laboratory tests for thyroid function and glycemic control, ensured the reliability and accuracy of our data.

However, our study also encountered several limitations that warrant consideration. Firstly, the non-probability sampling method employed may have introduced selection bias, potentially limiting the generalizability of our findings to broader populations of T2DM patients. Moreover, the observational nature of the study precludes establishment of causal relationships between glycemic control and thyroid dysfunction, highlighting the need for prospective longitudinal studies to validate our findings over time. Despite these limitations, our study contributes valuable insights into the prevalence and clinical implications of thyroid disorders among T2DM patients, paving the way for future research in this critical area of endocrinology.

Based on our findings, we recommend integrating routine thyroid function testing into the clinical management of type 2 diabetes mellitus (T2DM) patients, particularly those with suboptimal glycemic control. Early detection of thyroid disorders, indicated by abnormal TSH levels, could facilitate timely intervention and potentially improve patient outcomes. Clinicians should consider thyroid function tests as part of regular health assessments for T2DM patients, alongside standard glycemic monitoring, to ensure comprehensive management and reduce the risk of undiagnosed thyroid dysfunction complicating diabetes care.^25-27^

The findings of our study may be generalized cautiously to similar populations of T2DM patients, particularly those accessing tertiary care settings in similar geographic regions. However, variations in demographic characteristics, healthcare practices, and environmental factors across different populations could influence the prevalence and clinical manifestations of thyroid disorders. Future research should strive to include diverse patient cohorts to improve external validity and broaden the applicability of findings to a global context.^28,29^

Future research should focus on addressing several gaps identified in this study. Longitudinal studies are needed to explore the temporal relationship between glycemic control and thyroid dysfunction, shedding light on whether poor glycemic control precedes or exacerbates thyroid disorders in T2DM patients. Further understanding of common pathophysiological mechanisms may be gained through research examining the biological pathways that connect thyroid dysfunction and diabetes. Additionally, comparative studies across diverse populations and settings are essential to enhance the generalizability of findings and understand how demographic, geographic, and cultural factors influence the prevalence and clinical implications of thyroid disorders in T2DM.

## CONCLUSIONS

Our study reveals a high prevalence of thyroid disorders among individuals with type 2 diabetes mellitus (T2DM), with subclinical hypothyroidism being the most common manifestation. Importantly, we found that poorly controlled blood glucose levels (HbA1c ≥ 7.5) significantly increase the likelihood of thyroid dysfunction. Early detection and intervention can potentially mitigate the impact of thyroid disorders on diabetes outcomes. Moving forward, integrating thyroid function tests into regular diabetes care protocols could enhance clinical management and improve health outcomes for individuals living with T2DM.
